# 214 Moving Forward - Exercise Promotion At A Major Trauma Centre

**DOI:** 10.1093/eurpub/ckae114.138

**Published:** 2024-09-26

**Authors:** Ruairi Connolly, Eanna MacSuibhne, Sinead Long, Michael Dunphy, Stephen Halpin

**Affiliations:** Cork University Hospital, Ireland; Cork University Hospital, Ireland; Cork University Hospital, Ireland; Cork University Hospital, Ireland; Cork University Hospital, Ireland

## Abstract

**Purpose:**

Returning to exercise after traumatic injuries is essential. Regular movement plays a crucial role in rehabilitation and long-term health. Returning to exercise post trauma can be difficult due to new impairment, pain and fear of re-injury. With our initiative – Moving Forward – we aimed to integrate exercise promotion into care provision at a major trauma centre. The goal was to empower patients to exercise by providing them with personalised exercise programmes, information and peer support from inclusive sports organisations in their communities.

**Project Description:**

Development: Patients required insight into their injuries and personalised exercise programmes to support their rehabilitation. Collaborating closely with trauma facing services we developed a trauma care booklet. Each booklet was personalised to the patient and provided insights into their injuries as well as a tailor made exercise programme. The booklet highlighted the benefits of exercise, personalised exercise progressions and named contacts for peer support networks and sports communities in their region. A networking exercise facilitated identification of inclusive exercise resources. Booklet design was supported by an innovation designer.

**Implementation:**

A key worker was nominated for each patient who provided the trauma care booklet. Early provision of this document allowed the patient time to implement exercises in a supported environment. Exercise programmes were personalised and designed in collaboration with a Rehabilitation Medicine Consultant and trauma physiotherapists. Patients also availed of trauma psychology support. Liaison with suitable sports organisations and community services were made prior to hospital discharge.

**Evaluation:**

Patient outcomes, satisfaction and adherence to exercise programs were assessed. Feedback was incorporated to refine and enhance our services, ensuring it remained responsive to the diverse needs of our patients.

**Dissemination:**

Our experiences and methodologies are being shared through conferences and collaborative networks to inspire similar initiatives and foster a culture of exercise promotion within trauma care settings globally.

**Conclusions:**

Integrating exercise promotion into trauma care is possible. Empowering patients with personalised exercise programmes and peer support facilitates long term adherence to exercise. Fostering links with inclusive sports organisations can support patients to pursue sports and exercise long after discharge from a major trauma centre.

